# Supplementary material for: Differential Expression and Clinical Relevance of C-X-C Motif Chemokine Receptor 4 (CXCR4) in Renal Cell Carcinomas, Benign Renal Tumors, and Metastases
Source: Int J Mol Sci. 2023 Mar 9;24(6):5227. doi: 10.3390/ijms24065227 (PMC10048828; doi:10.3390/ijms24065227)
Supplement: Supplementary file 1 [file ijms-24-05227-s001.zip › ijms-2104411-supplementary.pdf]

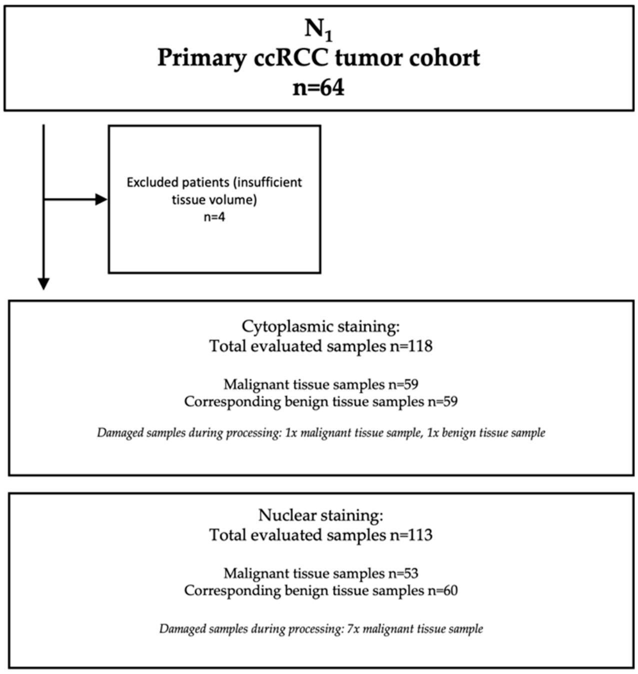

Supplementary Figure S1: Distribution of specimens evaluated for CXCR4 expression in cohort n_1. Abbreviations: ccRCC: clear cell renal cell carcinoma.

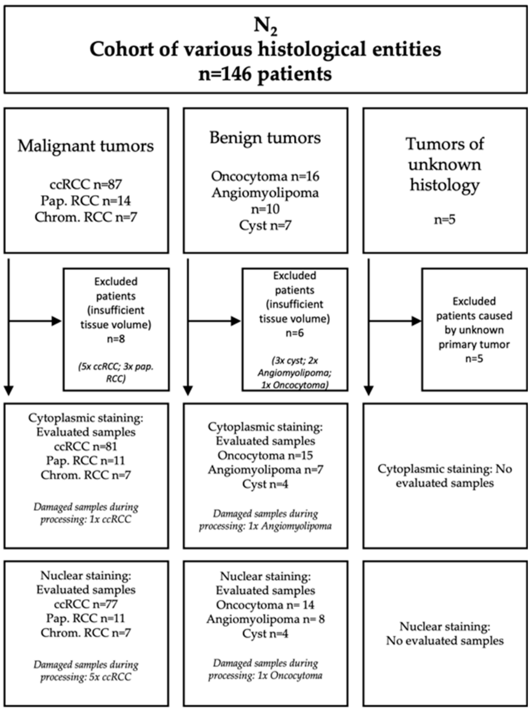

Supplementary Figure S2: Distribution of specimens evaluated for CXCR4 expression in cohort n_2. Abbreviations: ccRCC: clear cell renal cell carcinoma; pap. RCC: papillary renal cell carcinoma; chrom. RCC: chromophobic renal cell carcinoma.

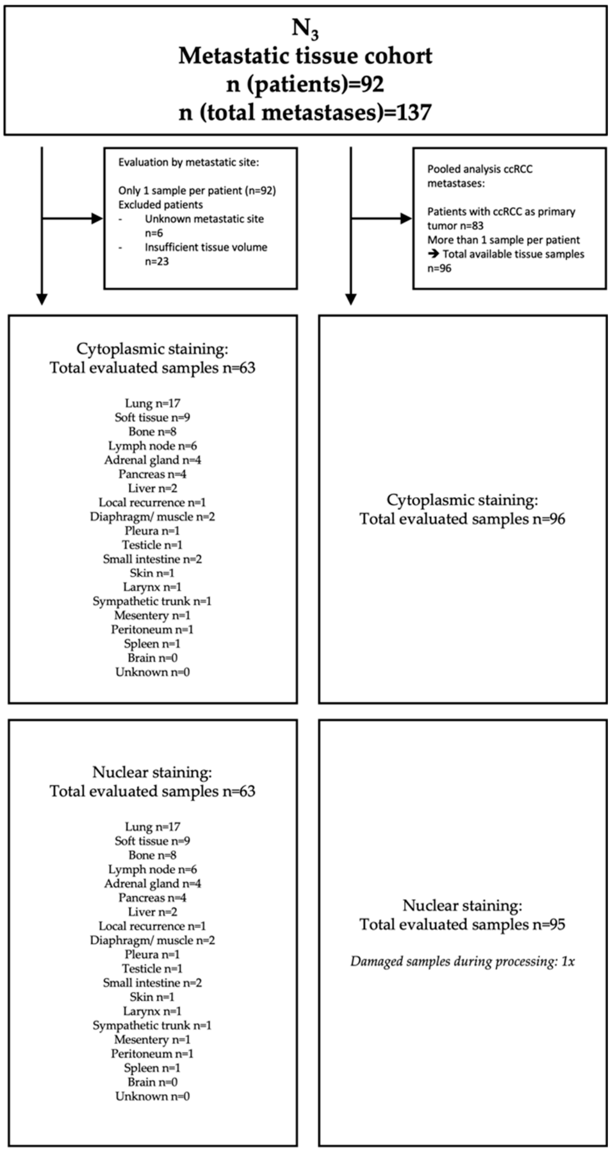

Supplementary Figure S3: Distribution of specimens evaluated for CXCR4 expression in cohort n3.

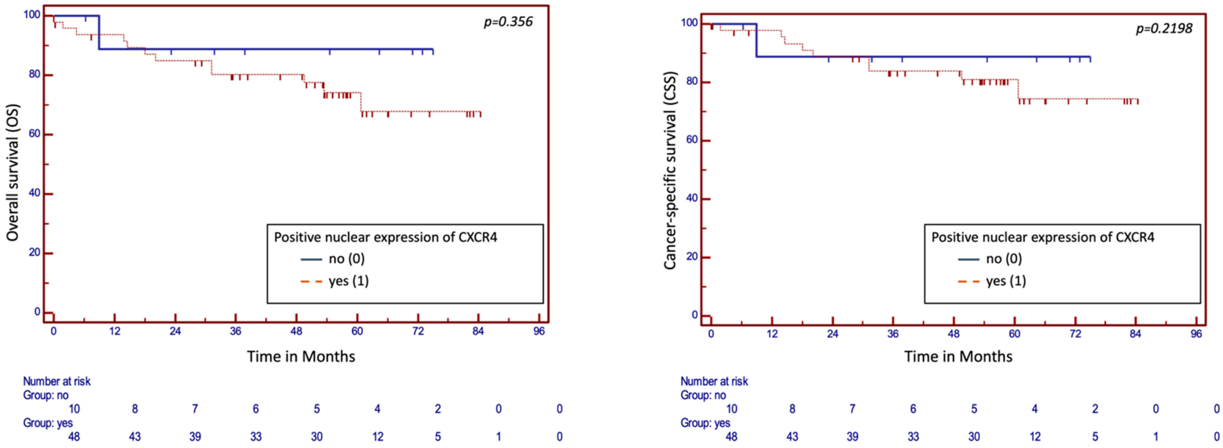

Supplementary Figure S4: Kaplan-Meier analysis for cohort n1: Overall Survival (OS) and cancer-specific survival (CSS) in dependence of positive nuclear CXCR4 expression.
